# Supplementary material for: Liquidity effects on oil volatility forecasting: From fintech perspective
Source: PLoS One. 2021 Nov 29;16(11):e0260289. doi: 10.1371/journal.pone.0260289 (PMC8629280; doi:10.1371/journal.pone.0260289)
Supplement: Attachment — Submitted filename: PONE-D-21-17939_report.pdf [file pone.0260289.s001.pdf]

Comments on manuscript PONE-D-21-17939 entitled "Liquidity Effects on Oil Volatility Forecasting: From Fintech Perspective"

This paper proposed a liquidity-adjusted GARCH model, denoted by LGARCH, for volatility forecasting by employing the Genetic Programming (GP) technique. An empirical study is conducted to investigate the fitting and prediction performances of the LGARCH model compared with the GARCH, IGARCH, and TGARCH models. The authors claimed that the LGARCH model overwhelmingly surpasses all the three GARCH models in both in-sample volatility fitting and out-of-sample volatility forecasting.

I agree that incorporating AI techniques is an exciting direction for modeling financial time series. This study belongs to this direction and focuses on including liquidity effects on oil volatility forecasting. My comments are as follows:

1. Since volatility cannot be observed directly, this study employs the SRV_t in (1) (or RV_t in (2)) as a proxy of volatility. The objective of the GP introduced in Section 3.2 is designed to minimize the error between the SRV_t (or RV_t) and the associated predicted variance. This design is highly related to the evaluation measure MSE_T defined in Section 4, where the $Observed_t$ is the SRV_t (or RV_t) and the $Predicted_t$ is the predicted variance.

   However, for the three considered GARCH models, the model parameters are estimated under different criteria. For example, they aim to minimize the L_2 loss between the observed returns and the associated predicted returns. In other words, the three GARCH models are not designed to produce volatilities close to SRV_t (or RV_t) but to capture the heteroscedasticity in the return process. The role of the volatility obtained from the three GARCH models is the conditional variance of r_t

conditional on the information up to $t-1$. Therefore, it is not surprising that their volatilities are not close to SRV_t (or RV_t).

Due to these facts, I would say that the comparison study in this article is not fair, and the conclusion that "the LGARCH model overwhelmingly surpasses all the three GARCH models" is not proper since the LGARCH and the three GARCH models are designed for different targets. Most importantly, the current comparison is difficult to identify whether the good performance of the LGARCH model is due to the liquidity effects or just because of the different estimation targets of the LGARCH and the three GARCH models.

2. In the literature, many other approaches are designed for estimating proxies of volatility similar to SRV_t (or RV_t), but without considering liquidity effects. For example, Chen et al. (2010) proposed a GARCH-SVM approach for volatility forecasting and adopted a proxy of volatility similar to RV_t. Hajizadeh et al. (2012) proposed an ANN-GARCH type model for forecasting the volatility of S&P 500 index return and employed a proxy of volatility similar to RV_t. I suggest the authors design a new comparison scenario to investigate whether the LGARCH outperforms these types of approaches and whether including the liquidity effects improves the volatility forecasting.

3. Many notations and relationships between random variables are not well defined. Some examples are listed in the following:

   (a) Line 151: P_t is not defined.

   (b) Line 151 and 169: Do P_t and S_t represent the same thing?

   (c) Line 167: η_t is not defined.

   (d) Line 167 and 169: The relationship between η_t and Q_t is not clear. Are they independent or uncorrelated?

(e) Line 176: How to estimate the '*spread*' from data is not clear.

(f) Line 178: The 'residual term' mentioned here is not clearly defined.

(g) Line 245: z_t is not defined.

(h) Line 254 and 255: y_{t-1} is not defined. If y_t denotes the day-t return, I suggest to replace it by r_t as in (1) and (2) for consistency purposes.

According to the above reasons, I do not recommend the manuscript for publication in its current state.

References

[1] Chen, S., Härdle, W. K., & Jeong, K. (2010). Forecasting volatility with support vector machine-based GARCH model. Journal of Forecasting, 29(4), 406-433.

[2] Hajizadeh, E., Seifi, A., Zarandi, M. F., & Turksen, I. B. (2012). A hybrid modeling approach for forecasting the volatility of S&P 500 index return. Expert Systems with Applications, 39(1), 431-436.
