# Supplementary material for: Liquidity effects on oil volatility forecasting: From fintech perspective
Source: PLoS One. 2021 Nov 29;16(11):e0260289. doi: 10.1371/journal.pone.0260289 (PMC8629280; doi:10.1371/journal.pone.0260289)
Supplement: Attachment — Submitted filename: Plosone_17939.pdf [file pone.0260289.s002.pdf]

# Report on the paper

## Liquidity Effects on Oil Volatility Forecasting: From Fintech Perspective

By Shusheng Ding, Tianxiang Cui, Yongmin Zhang, and Mohamed Shaban

### General summary

In order to forecast the oil volatility with liquidity, the authors propose the Genetic programming (GP) method to identify the model format. Based on the GP method, the authors propose the liquidity-adjusted GARCH (LGARCH) model. For comparison, they consider the GARCH, IGARCH, and GJR-GARCH models. The empirical results show that the proposed LGARCH model outperforms in both sample fitting and out of sample forecasting. The robust checking is guaranteed using the loss function $QLIKE$. The results are interesting but the authors need to revise the paper carefully such as undefined random errors or some functions. The comments and questions are as follows. In the hope that they will be helpful to the authors.

### Comments

1. In the GP method, the authors should explain the method in details. What is the criterion for choosing the best model? For example, in line 226, the definition of $r^2$ should be clarified. The selection basis should be clarified. For instance, in equation (6), is there any regular condition for the function $f$? Is there any penalty function to avoid the overfitting problem?

2. In empirical analysis, the authors could explain the results in details. For example, in Table 5, the MSE for the out of sample forecasting with simplified realized variance improve around 58% in 2012 but improve only 2% in 2013. On the other hand, the MSE for the out of sample forecasting with realized variance improve improves 98% in 2013 but only improves around 40% in 2014. Is there any significant difference between the above data?

3. In addition, in empirical studies, do the authors consider the rolling method for the estimation? The authors should clarify this.

4. In equation (3), (10), and (11), the definition of $\eta_t$ is missing. The authors should clarify this.

5. On page 5, the sentence from line 171 to line 173 "He further assumes that Qt is equally likely to be 171 +1 first difference of equation and plugs in the result from equation" is not clear. The authors should revise this.

6. On page 5, is $COV$ identical to $cov$? The notations have to be consistent.

7. On page 7, in line 245, $z_t$ is not defined clearly.

8. In equation (14), the authors propose the loss function $QLIKE$. However, the definition is not clear. The robustness implies that the term $\frac{\sigma_{t,j}}{\hat{\sigma}_{t,j}}$ is close to one but there is no constraint for the first term $\ln \hat{\sigma}_{t,j}$. Hence, the authors should clarify why the smaller $QLIKE$, the better performance.

9. Some notations must be Italic such as in line 170 "$E$" and in line 350 and line 351 "$t$."
