# Supplementary material for: Liquidity effects on oil volatility forecasting: From fintech perspective
Source: PLoS One. 2021 Nov 29;16(11):e0260289. doi: 10.1371/journal.pone.0260289 (PMC8629280; doi:10.1371/journal.pone.0260289)
Supplement: Attachment — Submitted filename: Response to Reviewers.pdf [file pone.0260289.s003.pdf]

Response to Reviewers

Comments from Reviewer 1

1. In the GP method, the authors should explain the method in details. What is the criterion for choosing the best model? For example, in line 226, the definition of r^2 should be clarified. The selection basis should be clarified. For instance, in equation (6), is there any regular condition for the function f? Is there any penalty function to avoid the overfitting problem?

   Response: Thanks, the selection criterion has been further clarified. Normally, the ending conditions for evolutionary computation based method are a desired solution is found (the measure of function fitness approximates 0), or the maximum number of generations is reached. For this work, as the fitness measure will never reach 0, therefore the system will terminate after a certain pre-defined number of generations (100 in this work). The parameters settings used here are based on empirical experiences and we do not claim that these settings are the optimal choices. Our main purpose is to test the effectiveness of GP approach on volatility forecasting task. r^2 can be defined as the squared return term, which is used to approximate the volatility. We do not use the regular condition for function f, in order to prevent overfitting, we further eliminate the forecasting functions with more than 10 terms obtained by GP, which is similar to the idea of dropout in neural network training.

2. In empirical analysis, the authors could explain the results in details. For example, in Table 5, the MSE for the out of sample forecasting with simplified realized variance improve around 58% in 2012 but improve only 2% in 2013. On the other hand, the MSE for the out of sample forecasting with realized variance improve improves 98% in 2013 but only improves around 40% in 2014. Is there any significant difference between the above data?

   Response: Nevertheless, for the out-of-sample forecasting, the results may not be as stable as the in sample fitting. For the data of 2013 and 2014, the oil price move trend has been changed. In 2013, the oil price had an increasing trend with the annual return of 6.90%. On the other hand, however, the oil price plummeted in 2014, with the annual return of -45.55%, which yielded the lowest price of $53.45 since 2010. As a result, the return volatility movement may not follow similar pattern, which may result in the significant difference of volatility forecasting.

3. In addition, in empirical studies, do the authors consider the rolling method for the estimation? The authors should clarify this.

   Response: we have considered the rolling over method. For the out-of-sample forecasting, we use previous years data to forecast the volatility of this year. For example, we use the data of 2013 to estimate the model parameters and then use those parameters to forecast volatility of year 2014. Similarly, we use the data of 2014 to estimate the model parameters and then use those parameters to forecast volatility of year 2015. We thereby roll over the sample by one year.

4. In equation (3), (10), and (11), the definition of η_t is missing. The authors should clarify this.

   Response: Thanks, η_t has now been defined.

5. On page 5, the sentence from line 171 to line 173 "He further assumes that Q_t is equally likely to be 171 +1 first difference of equation and

plugs in the result from equation" is not clear. The authors should revise this.

Response: Thanks, we have corrected this.

6. On page 5, is COV identical to cov? The notations have to be consistent.

   Response: Thanks, we have corrected this.

7. On page 7, in line 245, z_t is not defined clearly.

   Response: Thanks, z_t has now been defined.

8. In equation (14), the authors propose the loss function QLIKE. However, the definition is not clear. The robustness implies that the term $\frac{\sigma_{t,j}}{\hat{\sigma}_{t,j}}$ is close to one but there is no constraint for the first term $\ln \hat{\sigma}_{t,j}$. Hence, the authors should clarify why the smaller QLIKE, the better performance.

   Response: Thanks, to further justify the issue with QLIKE, we adopted the normalize loss function for QLIKE (see equation (16)), which is mainly used for out-of-sample test.

9. Some notations must be Italic such as in line 170 "E" and in line 350 and line 351 "t".

   Response: Thanks, we have corrected this.

Comments from Reviewer 2

1. Since volatility cannot be observed directly, this study employs the SRV_t in (1) (or RV_t in (2)) as a proxy of volatility. The objective of the GP introduced in Section 3.2 is designed to minimize the error between the SRV_t (or RV_t) and the associated predicted variance. This design is highly related to the evaluation measure MSE_T defined in Section 4, where the Observed t is the SRV_t (or RV_t) and the $Predicted_t$ is the predicted variance. However, for the three considered GARCH

models, the model parameters are estimated under different criteria. For example, they aim to minimize the L_2 loss between the observed returns and the associated predicted returns. In other words, the three GARCH models are not designed to produce volatilities close to SRV_t (or RV_t) but to capture the heteroscedasticity in the return process. The role of the volatility obtained from the three GARCH models is the conditional variance of r_t conditional on the information up to $t-1$. Therefore, it is not surprising that their volatilities are not close to SRV_t (or RV_t).

Due to these facts, I would say that the comparison study in this article is not fair, and the conclusion that "the LGARCH model overwhelmingly surpasses all the three GARCH models" is not proper since the LGARCH and the three GARCH models are designed for different targets. Most importantly, the current comparison is difficult to identify whether the good performance of the LGARCH model is due to the liquidity effects or just because of the different estimation targets of the LGARCH and the three GARCH models.

Response: There are two reasons why the comparison is fair. Firstly, although the GARCH model is designed to capture the heteroscedasticity in the return process, the purpose of capturing the heteroscedasticity is to accurately forecast the volatility. The heteroscedasticity means the volatility of the random variable varies with time. The GARCH model is designed to capture such volatility movement pattern in order to predict its future path. More importantly, our model is based on GARCH model and the format is quite similar with the original GARCH model, which cannot have huge estimation target difference compared with the original GARCH model.

Furthermore, for the purpose of capturing the dynamics of volatility, the family of GARCH models has been widely applied to energy markets such as oil market. Sadorsky (2006) [5]for instance, finds that the

threshold GARCH (or GJR, Glosten et al., 1993[1]) fits well for heating oil and natural gas volatilities, whereas the standard GARCH(1,1) model fits well for crude oil and unleaded gasoline volatilities. Kang et al. (2009)[2] show that component GARCH (CGARCH) and fractional integrated GARCH (FIGARCH) provide superior performance. Recently, Ma et al. (2021)[4] prove GARCH family models fit oil volatility quite smoothly. Liang et al. (2021)[3] also demonstrate that GARCH-MIDAS-type models provide solid performance for natural gas futures volatility forecasting.

2. In the literature, many other approaches are designed for estimating proxies of volatility similar to SRV_t (or RV_t), but without considering liquidity effects. For example, Chen et al. (2010) proposed a GARCH-SVM approach for volatility fore- casting and adopted a proxy of volatility similar to RV_t . Hajizadeh et al. (2012) proposed an ANN-GARCH type model for forecasting the volatility of S&P 500 index return and employed a proxy of volatility similar to RV_t . I suggest the authors design a new comparison scenario to investigate whether the LGARCH outperforms these types of approaches and whether including the liquidity effects improves the volatility forecasting.
   Response: Thanks, we have now included the ANN-GARCH and GARCH-SVM model comparison in our robustness check part.

3. Many notations and relationships between random variables are not well defined. Some examples are listed in the following:
   (a) Line 151: P_t is not defined.
   Response: Thanks, P_t has now been defined.
   (b) Line 151 and 169: Do P_t and S_t represent the same thing?
   Response: Not exactly, P_t is the last day's settlement price and S_t is last observed traded price.

(c) η_t is not defined.

Response: Thanks, η_t has now been defined.

(d) Line 167 and 169: The relationship between η_t and Q_t is not clear. Are they independent or uncorrelated?

Response: Thanks, the relationship has now been defined.

(e) How to estimate the 'spread' from data is not clear.

Response: Thanks, it has been clarified now.

(f) The 'residual term' mentioned here is not clearly defined.

Response: Thanks, the 'residual term' has been clarified now.

(g) z_t is not defined.

Response: Thanks, z_t has been defined now.

(h) Line 254 and 255: y_{t-1} is not defined. If y_t denotes the $day - t$ return, I suggest to replace it by r_t as in (1) and (2) for consistency purposes.

Response: Thanks, y_{t-1} has now been defined. y_t denotes the residual term, which contains the noise information that has not been captured by the GARCH-X model, and it is different from r_t.

Additional Comments from Editor

1. The authors use the period from January 1, 2001 to December 31, 2010 as the in-sample period and the period from January 1, 2011 to December 31, 2016 as the out-of-sample period. However, the data is too old. The authors are recommended to extend the out-of-sample period to 2019 at least and to update the results.

   Response: Thanks, we have updated our results to the year of 2019.

2. The authors should normalize both loss functions, MSE and QLIKE, to be the robust and homogeneous loss functions proposed by Patton (2011); see Chen and Watanabe (2019) for details.

Response: Thanks, we have used the normalize loss functions, for both MSE and QLIKE.

References

[1] Lawrence R Glosten, Ravi Jagannathan, and David E Runkle. On the relation between the expected value and the volatility of the nominal excess return on stocks. *The journal of finance*, 48(5):1779–1801, 1993.

[2] Sang Hoon Kang, Sang-Mok Kang, and Seong-Min Yoon. Forecasting volatility of crude oil markets. *Energy Economics*, 31(1):119–125, 2009.

[3] Chao Liang, Feng Ma, Lu Wang, and Qing Zeng. The information content of uncertainty indices for natural gas futures volatility forecasting. *Journal of Forecasting*, 2021.

[4] Feng Ma, Chao Liang, Qing Zeng, and Haibo Li. Jumps and oil futures volatility forecasting: a new insight. *Quantitative Finance*, 21(5):853–863, 2021.

[5] Perry Sadorsky. Modeling and forecasting petroleum futures volatility. *Energy Economics*, 28(4):467–488, 2006.
