# Supplementary material for: Liquidity effects on oil volatility forecasting: From fintech perspective
Source: PLoS One. 2021 Nov 29;16(11):e0260289. doi: 10.1371/journal.pone.0260289 (PMC8629280; doi:10.1371/journal.pone.0260289)
Supplement: Attachment — Submitted filename: Plosone_17939_R1.pdf [file pone.0260289.s004.pdf]

Report on the paper

Liquidity Effects on Oil Volatility Forecasting: From Fintech Perspective R1

By Shusheng Ding, Tianxiang Cui, Yongmin Zhang, and Mohamed Shaban

General summary

The paper is now well written. However, there are still several comments as follows.

Comments

1. In line 247, the authors eliminates the forecasting functions with more than 10 terms to prevent the overfitting. The statement is not very clear. First, does this mean that the GP method contains the estimation part and the forecasting part? It could be more clear if the authors can explain how to balance these two parts briefly. Second, the authors how to verify that 10 is the best choice for overfitting. Is there any optimization criterion?

2. In line 368, the authors give the discussion for the instability for the out-of-sample forecasting saying that the volatility movement may not follow similar pattern. Is it possible to overcome this difficulty such as remove more forecasting terms in the GP method.

3. In line 182, *cov* should be cov.

4. In line 175, t should be t.
