# Supplementary material for: Liquidity effects on oil volatility forecasting: From fintech perspective
Source: PLoS One. 2021 Nov 29;16(11):e0260289. doi: 10.1371/journal.pone.0260289 (PMC8629280; doi:10.1371/journal.pone.0260289)
Supplement: Attachment — Submitted filename: PONE-D-21-17939R1_report.pdf [file pone.0260289.s005.pdf]

Comments on manuscript PONE-D-21-17939R1 entitled "Liquidity Effects on Oil Volatility Forecasting: From Fintech Perspective"

This revision does not satisfactorily address some of my previous questions.

1. The authors only mentioned that they added ANN-GARCH and SVM-GARCH methods for comparison in a paragraph before Section 5, but did not provide a brief introduction, the procedures, and related references of these two methods.

2. Line 172: The definition of η_t is still unclear. Is $\{\eta_t, t = 1, 2, \ldots\}$ a white noise process? Or do η_t's follow any specific distribution?

3. Line 190: The 'residual term ε_t ...'. What is the definition of ε_t? One can estimate ε_t from which equation before Line 190. In addition, the η_t in eqn. (3) is also called 'the residual term'. I'm confused about which one is 'the residual term'.

4. Line 266: The definition of z_t is still incomplete. Are there any moment assumptions of z_t?

5. Line 275: Again, we have another 'the residual term', denoted by y_t. I suggest the authors to clarify the roles of η_t, ε_t and y_t in the manuscript.
