# Supplementary material for: Liquidity effects on oil volatility forecasting: From fintech perspective
Source: PLoS One. 2021 Nov 29;16(11):e0260289. doi: 10.1371/journal.pone.0260289 (PMC8629280; doi:10.1371/journal.pone.0260289)
Supplement: Attachment — Submitted filename: Response to Reviewers.pdf [file pone.0260289.s006.pdf]

Response to Reviewers

Comments from Reviewer 1

1. In line 247, the authors eliminate the forecasting functions with more than 10 terms to prevent the overfitting. The statement is not very clear. First, does this mean that the GP method contains the estimation part and the forecasting part? It could be more clear if the authors can explain how to balance these two parts briefly.

   Response: Thank you for your comments. We have further clarified this issue. GP does not contain the estimation part and the forecasting part. As the number of generation increases, the average error between the value obtained by forecasting functions and the target value may decrease. In this case, GP may produce some high-order terms in order to further reduce the error. However, these high-order terms may cause the overfitting problem, which we need to prevent. Therefore, we eliminate the forecasting functions with more than 10 terms.

2. Second, the authors how to verify that 10 is the best choice for overfitting. Is there any optimization criterion?

   Response: Thank you for your comments. The choice of 10 is based on our empirical experience. In fact, the balance between exploration (to generate completely different forecasting functions to explore the search space) and exploitation (to include some high-order terms in the current best forecasting function to further reduce the estimation error) is an open question. We do not claim 10 is the optimal choice.

Our main purpose is to test the effectiveness of GP approach on volatility forecasting task.

3. In line 368, the authors give the discussion for the instability for the out-of-sample forecasting saying that the volatility movement may not follow similar pattern. Is it possible to overcome this difficulty such as remove more forecasting terms in the GP method.

   Response: Thank you for your comments. In fact, this is a trade-off issue in the volatility forecasting area. It is true that the instability will decrease as you remove more forecasting terms in the GP method. Nevertheless, when you remove more terms in the GP method, the in-sample fitting becomes extremely difficult and solid forecasting model generation will be frustrated.

4. In line 182, *cov* should be cov.

   Response: Thank you for your comments, we have corrected this mistake.

5. In line 175, t should be t.

   Response: Thank you for your comments, we have corrected this mistake.

Comments from Reviewer 2

1. The authors only mentioned that they added ANN-GARCH and SVM-GARCH methods for comparison in a paragraph before Section 5, but did not provide a brief introduction, the procedures, and related references of these two methods.

   Response: Thank you for your comments. Now we have included a brief introduction, the procedures of ANN-GARCH and SVM-GARCH with references. The ANN-GARCH model is based on the Artificial Neural Networks, which is the non-parametric method. This method

can be applied to uncover the nonlinear associations between the parameters of the GARCH model. Nevertheless, ANN-GARCH is less flexible compared with other models such as neural fuzzy inference system. In addition, the backpropagation algorithm of the ANN disables itself to learn from its own forecasting error. (Kristjanpoller and Minutolo, 2016 [5]). For the ANN-GARCH method, each neural network connects a group of volatility forecasting variables with output variables and a number of hidden layers. Neurons are connected between the layers for connections that are activated by triggering a threshold. The input group of variables and output group of variables can be a combination of all of different number of neurons in each layer, where the relations between inputs and outputs are embedded (see Kristjanpoller and Hernndez, 2017 [4]; Bhattacharya and Ahmed, 2018 [1]). The SVM-GARCH model is based on the support vector machine (SVM) stems from the statistical learning theory (see Cortes and Vapnik, 1995 [3]), For the SVM method, it develops a nonlinear mapping function from input space towards a high-dimensional hidden space. On the basis, SVM estimates a linear regression model in the output space, which corresponds to a nonlinear regression in the low-dimensional input space. Theoretically, SVM could approximate any nonlinear mapping relations between input space and output space. Nevertheless, since SVM has different kernels, for instance, linear kernel, polynomial kernel, and Gaussian kernel, the choice of kernels may become tricky. For example, if the residuals of regression model are Gaussian, the results that SVM delivers may not be solid enough, since this kernel is based on Probability Distribution Function, residuals generated by SVM may not follow Gaussian distribution. (see Perez-Cruz et al., 2003 [6]; Chen et al., 2010 [2]).

2. Line 172: The definition of η_t is still unclear. Is $\{\eta_t, t = 1, 2, \ldots\}$ a white noise process? Or do η_t's follow any specific distribution?

Response: Thank you for your comments, η_t is the mean-zero, serially uncorrelated public information shock on day t.

3. Line 190: The 'residual term $\varepsilon_t \ldots$'. What is the definition of ε_t? One can estimate ε_t from which equation before Line 190. In addition, the η_t in eqn. (3) is also called 'the residual term'. I'm confused about which one is 'the residual term'.

   Response: Thank you for your comments, they are both the residual term for different time series models. η_t is the residual term for fundamental value model, based on eqn. (3) and ε_t is the residual term for GARCH model (see eqn. (8)), which is:

$$\begin{aligned} \sigma_t^2 &= \alpha_0 + \alpha_1 \sigma_{t-1}^2 + \alpha_2 \varepsilon_{t-1}^2, \\ r_t &= \phi + \varepsilon_t, \end{aligned} \tag{1}$$

   where σ_t is the volatility of target time series and ε_t is the residual term from the return prediction equation, ϕ is the conditional mean, and $\varepsilon_t \sim N(0, \sigma_t^2)$.

4. The definition of z_t is still incomplete. Are there any moment assumptions of z_t?

   Response: Thank you for your comments, z_t is the noise term, which is defined as $z_t \sim IID(0, 1)$.

5. Line 275: Again, we have another 'the residual term', denoted by y_t. I suggest the authors to clarify the roles of η_t, ε_t and y_t in the manuscript.

   Response: Thank you for your comments. We have clarified all three terms now and they are all the residual term for different time series models.

   - η_t is the residual term for fundamental value model, based on eqn. (3).

- ε_t is the residual term for GARCH model (see eqn. (8)), which is:

$$\begin{aligned}\sigma_t^2 &= \alpha_0 + \alpha_1\sigma_{t-1}^2 + \alpha_2\varepsilon_{t-1}^2,\\ r_t &= \phi + \varepsilon_t,\end{aligned} \tag{2}$$

where σ_t is the volatility of target time series and ε_t is the residual term from the return prediction equation, which is: ϕ is the conditional mean, and $\varepsilon_t \sim N(0, \sigma_t^2)$.

- y_t is the residual term for GARCH-X model (see eqn. (9)). In particular, $y_t^2 = \sigma_t^2 * z_t^2$, where σ_t is the volatility of target time series and z_t is the noise term, which is defined as $z_t \sim IID(0, 1)$.

Additional Comments from Editor

1. All values shown in Tables 3-6 are extremely small. This situation can be improved by rescaling the time series $\{r_t\}$. Researchers use the following formula for the return. Line 156: $R_t = (ln(p_t) - ln(p_{t-1})) \times 100$.

   Response: Thank you for your comments and we have rescaled the returns which helps us to avoid extremely small values.

2. Eq. (2) should be rescaled too. Most related papers adopt the following formula for realized volatility in order to avoid small values. $RV*_t = (RV_t \times 10,000)$ or $RV**_t = ln(RV_t \times 10,000)$.

   Response: Thank you for your comments and we have rescaled the volatility estimation for both eqn. (1) and eqn. (2).

References

[1] Sujoy Bhattacharya and Arshad Ahmed. Forecasting crude oil price volatility in india using a hybrid ann-garch model. *International Journal of Business Forecasting and Marketing Intelligence*, 4(4):446–457, 2018.

[2] Shiyi Chen, Wolfgang K Härdle, and Kiho Jeong. Forecasting volatility with support vector machine-based garch model. *Journal of Forecasting*, 29(4):406–433, 2010.

[3] Corinna Cortes and Vladimir Vapnik. Support-vector networks. *Machine learning*, 20(3):273–297, 1995.

[4] Werner Kristjanpoller and Esteban Hernández. Volatility of main metals forecasted by a hybrid ann-garch model with regressors. *Expert Systems with Applications*, 84:290–300, 2017.

[5] Werner Kristjanpoller and Marcel C Minutolo. Forecasting volatility of oil price using an artificial neural network-garch model. *Expert Systems with Applications*, 65:233–241, 2016.

[6] Fernando Pérez-Cruz, Julio A Afonso-Rodriguez, and Javier Giner. Estimating garch models using support vector machines. *Quantitative Finance*, 3(3):163, 2003.
