# Supplementary material for: Liquidity effects on oil volatility forecasting: From fintech perspective
Source: PLoS One. 2021 Nov 29;16(11):e0260289. doi: 10.1371/journal.pone.0260289 (PMC8629280; doi:10.1371/journal.pone.0260289)
Supplement: Attachment — Submitted filename: Plosone_17939_R2.pdf [file pone.0260289.s007.pdf]

Report on the paper

Liquidity Effects on Oil Volatility Forecasting: From Fintech Perspective R1

By Shusheng Ding, Tianxiang Cui, Yongmin Zhang, and Mohamed Shaban

General summary

There is still one more issue for the paper.

Comments

1. Based on the advice, the definition of r_t should be

   $$r_t = \log\left(\frac{P_t}{P_{t-1}}\right) \times 100.$$

   However, the authors propose

   $$r_t = \log\left(\frac{P_t}{P_{t-1}}\right) \times 10000.$$

   The authors should clarify this.
