# Supplementary material for: Liquidity effects on oil volatility forecasting: From fintech perspective
Source: PLoS One. 2021 Nov 29;16(11):e0260289. doi: 10.1371/journal.pone.0260289 (PMC8629280; doi:10.1371/journal.pone.0260289)
Supplement: Attachment — Submitted filename: PONE-D-21-17939R2_report.pdf [file pone.0260289.s008.pdf]

Comments on manuscript PONE-D-21-17939R2 entitled "Liquidity Effects on Oil Volatility Forecasting: From Fintech Perspective"

The authors have satisfactorily responded to all my questions in this revision. Nevertheless, I notice that the authors multiplied the values of SRV_t and RV_t by $10,000$ in eqn. (1) and (2), respectively, to avoid extreme small values in this revision. Accordingly, the numerical results in Tables 3-6 are also revised. In this part, I find one typo and have a minor comment.

1. Should $r_t = \ln(P_t/P_{t-1}) \times 10,000$ below eqn. (1) be replaced by $r_t = \ln(P_t/P_{t-1}) \times 100$?

2. In the captions of Tables 3-6, it will be better to replace the e.g. "$e - 06 = *10^{-6}$" by "$e - 02 = \times 10^{-2}$".
