# Supplementary material for: Liquidity effects on oil volatility forecasting: From fintech perspective
Source: PLoS One. 2021 Nov 29;16(11):e0260289. doi: 10.1371/journal.pone.0260289 (PMC8629280; doi:10.1371/journal.pone.0260289)
Supplement: Attachment — Submitted filename: Report_PONE-D-21-17939R2.pdf [file pone.0260289.s009.pdf]

To be clear and honest, I am not satisfied with the authors' response. The following two comments are from my previous suggestions. However, the authors do not follow my suggestions for the returns. We can see the range of returns is (-0.1654, 0.1640) in Table 1, which does not follow the formula given below.

Most papers in this area use "return" is defined as follows.

$$r_t = [\ln(p_t) - \ln(P_{t-1})] \times 100,\ t = 2, \ldots, n,$$

where p_t is the price of time t. The authors will have one last chance to revise it and all related results accordingly.

1) All values shown in Tables 3 - 6 are extremely small. This situation can be improved by rescaling the time series {r_t}. Researchers use the following formula for the return.

Line 156: R_t=(ln(p_t)-ln(p_{t-1}))\times 100.

2) Eq. (2) should be rescaled too. Most related papers adopt the following formula for realized volatility in order to avoid small values.

RV*_t= (RV_t \times 10,000) or

RV**_t=ln(RV_t \times 10,000).
