# Supplementary material for: Liquidity effects on oil volatility forecasting: From fintech perspective
Source: PLoS One. 2021 Nov 29;16(11):e0260289. doi: 10.1371/journal.pone.0260289 (PMC8629280; doi:10.1371/journal.pone.0260289)
Supplement: Attachment — Submitted filename: Response to Reviewers.pdf [file pone.0260289.s010.pdf]

Response to Reviewers

Comments from Editor

1. All values shown in Tables 3-6 are extremely small. This situation can be improved by rescaling the time series $\{r_t\}$. Researchers use the following formula for the return. Line 156: $R_t = (ln(p_t) - ln(p_{t-1})) \times 100$.

   Response: Thank you for your comments and we have rescaled the returns exactly according to your formula. Sorry for the mistake in the last manuscript.

2. Eq. (2) should be rescaled too. Most related papers adopt the following formula for realized volatility in order to avoid small values. $RV*_t = (RV_t \times 10,000)$ or $RV**_t = ln(RV_t \times 10,000)$.

   Response: Thank you for your comments and we have rescaled the volatility estimation exactly according to your formula for both eqn. (1) and eqn. (2). Sorry for the mistake in the last manuscript.

Comments from Reviewer 1

1. Based on the advice, the definition of r_t should be: $R_t = (ln(p_t) - ln(p_{t-1})) \times 100$. However, the authors propose: $R_t = (ln(p_t) - ln(p_{t-1})) \times 10000$. The authors should clarify this.

   Response: Thank you for your comments and we now followed the editor's comment where we define the $R_t = (ln(p_t) - ln(p_{t-1})) \times 100$.

Comments from Reviewer 2

1. Should $r_t = ln(P_t/P_{t-1}) \times 10000$ below eqn. (1) be replaced by $r_t = ln(P_t/P_{t-1}) \times 100$?

   Response: Thank you for your comments and we now followed the editor's comment where we define the $R_t = (ln(p_t) - ln(p_{t-1})) \times 100$.

2. In the captions of Tables 3-6, it will be better to replace the e.g. "$e - 06 = \times 10^{-6}$" by "$e - 02 = \times 10^{-2}$".

   Response: Thank you for your comments and we now replaced "$e - 06 = \times 10^{-6}$" by "$e - 02 = \times 10^{-2}$".
